# The association between copper transporters and the prognosis of cancer patients undergoing chemotherapy: a meta-analysis of literatures and datasets

**DOI:** 10.18632/oncotarget.13917

**Published:** 2016-12-12

**Authors:** Si Sun, Jing Cai, Qiang Yang, Simei Zhao, Zehua Wang

**Affiliations:** ^1^ Department of Obstetrics and Gynecology, Union Hospital, Tongji Medical College, Huazhong University of Science and Technology, Wuhan, Hubei, Peoples Republic of China

**Keywords:** CTR1, ovarian cancer, lung cancer, platinum-based chemotherapy, meta-analysis

## Abstract

Copper transporter 1 (CTR1), copper transporter 2 (CTR2), copper-transporting p-type adenosine triphosphatase 1 and 2 (ATP7A and ATP7B) are key mediators of cellular cisplatin, carboplatin and oxaliplatin accumulation. In this meta-analysis, we aimed to evaluate the relation of CTR1, CTR2, ATP7A and ATP7B to overall survival (OS), progression-free survival (PFS), disease-free survival (DFS) and treatment response (TR) of cancer patients who received chemotherapy based on published literatures, the Gene Expression Omnibus (GEO) and the Cancer Genome Atlas (TCGA) datasets. Hazard ratios (HRs) and odds ratios (ORs) were pooled using random-effect models. Subgroup analysis and sensitivity analysis were conducted; heterogeneity and publication bias were assessed. Twelve literatures and eight datasets with 2149 patients were included. Our results suggested that high CTR1 expression was associated with favorable OS, PFS, DFS and TR in cancer patients who underwent chemotherapy with acceptable heterogeneity. The relationship of CTR1 to cancer prognosis remained significant in the subgroup of patients who underwent platinum-based chemotherapy, the patients with ovarian cancer and those with lung cancer. The significance of these relationships was not influenced by geological region of publication, data origin or detection method. However, there was no evidence for relation of CTR2, ATP7A or ATP7B to OS, PFS, DFS or TR. Test of publication bias and sensitivity analysis suggested a robustness of all the summary effect estimates. In conclusion, high CTR1 level predicts prolonged survival and enhanced response to chemotherapy in cancer patients who underwent chemotherapy and CTR1 might be a potential target to circumvent chemotherapy resistance.

## INTRODUCTION

Platinum-based chemotherapy remains a first-line treatment of various epithelial malignancies including lung, bladder, head and neck and gynecological cancers for over 30 years. Although over half of the patients respond well to platinum-based chemotherapy, relapse and drug-resistance are major limitations for successful treatment of cancers. The platinum drugs exert cytotoxicity mainly through binding DNA to form crosslinks, which interfere normal DNA activities and induce cellular apoptosis [1]. However in resistant cancer cells, decreased cellular drug accumulation impairs the formation of DNA-platinum abducts. Low platinum concentration in tumor tissue is significantly associated with reduced tumor response [2, 3]. Therefore, drug accumulation is identified as a key process that governs response to chemotherapy in cancer.

**Figure 1:**
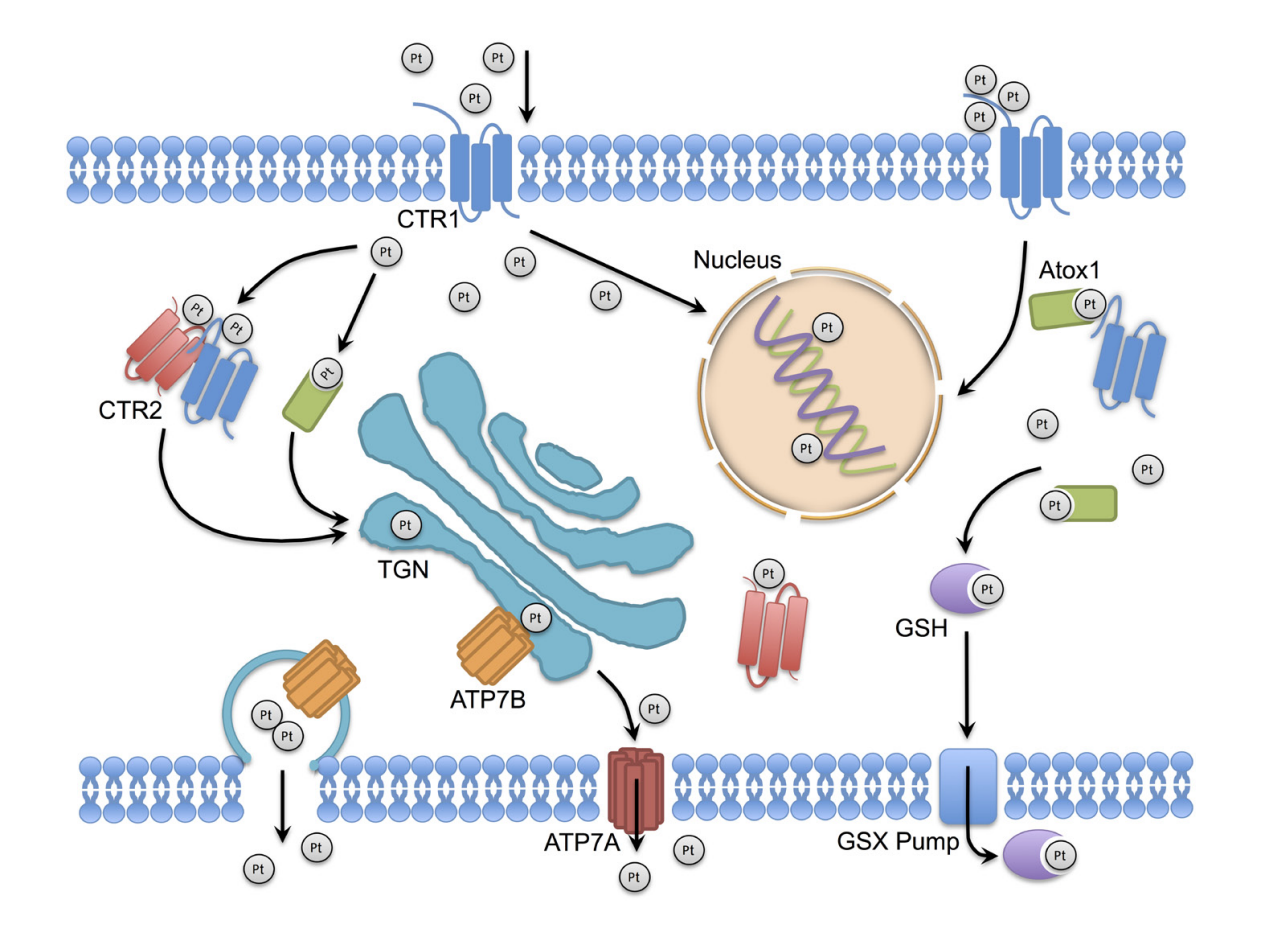
The role of copper homeostasis system in platinum drug transportation and trafficking Platinum drugs enter the cells by active CTR1 transportation. CTR1 and CTR2 ferry platinum to molecular chaperones, cell organelles and drug targets to proceeding platinum distribution or to initiate biological pathways (such as cytotoxic pathway and detoxication pathway). ATP7A and ATP7B regulate the subcellular distribution of cytosol platinum to trans Golgi network and mediate the removal of cellular platinum complex. CTR1, copper transporter 1; CTR2, copper transporter 2; ATP7A, copper-transporting p-type adenosine triphosphatases 1; ATP7B, copper-transporting p-type adenosine triphosphatases 2; TGN, trans Golgi network; GSH, glutathione; Atox1, human antioxidant protein 1; GS-X pump, glutathione S-conjugate pump.

Precedent evidence suggested that platinum drugs entered cancer cells through passive diffusion because the time-linear influx of platinum did not reach saturation at relevant drug concentration, was not inhibited by any structural analogous nor was it dependent on an optimal pH [4]. According to recent studies however, platinum accumulation was predominantly determined by transporters, which mediate either active or passive transportation of the drug [5]. Early evidence revealed that cellular cisplatin concentration was associated with cellular copper concentration [6, 7]. Studies from several independent laboratories revealed that copper metabolic pathways were involved in influx and efflux of platinum. CTR1 (copper transporter 1), CTR2 (copper transporter 2), ATP7A (copper-transporting p-type adenosine triphosphatase 1) and ATP7B (copper-transporting p-type adenosine triphosphatase 2) are four major copper transporters which mediate homeostasis and copper metabolism. With two conserved copper/cisplatin binding motifs, CTR1 facilitates copper and platinum to traverse through cellular membrane. CTR2 regulates copper/cisplatin storage by interacting and truncating CTR1 to smaller fragment, which is deprived of the ability to transport copper and cisplatin [8]. ATP7A sequesters intracellular platinum complex distribution and facilitates efflux of platinum complex. The turnover of ATP7B between trans Golgi network and cell membrane through endosomal/lysomal vesicle trafficking regulates cellular copper hemostasis by eliminating excess copper, which is also involved in cellular platinum elimination [9]. In addition to cisplatin, carboplatin and oxaliplatin are also substrates of these copper transporters [10–12] (Figure 1). Preclinical studies demonstrated that CTR1, CTR2, ATP7A and ATP7B expression regulate cellular response to cisplatin [8, 13–15]. However, the relationship between these copper transporters and the prognosis of cancer patients who received chemotherapy are inconclusive.

Therefore, we conducted a meta-analysis of published literatures as well as datasets to assess the association between the expression of copper transporters and the prognosis in cancer patients who underwent chemotherapy. To obtain more detailed information, subgroup analyses stratified by cancer type, chemotherapy regimen, geological region, data origin and detection method were conducted.

## RESULTS

### Study characteristics

**Figure 2:**
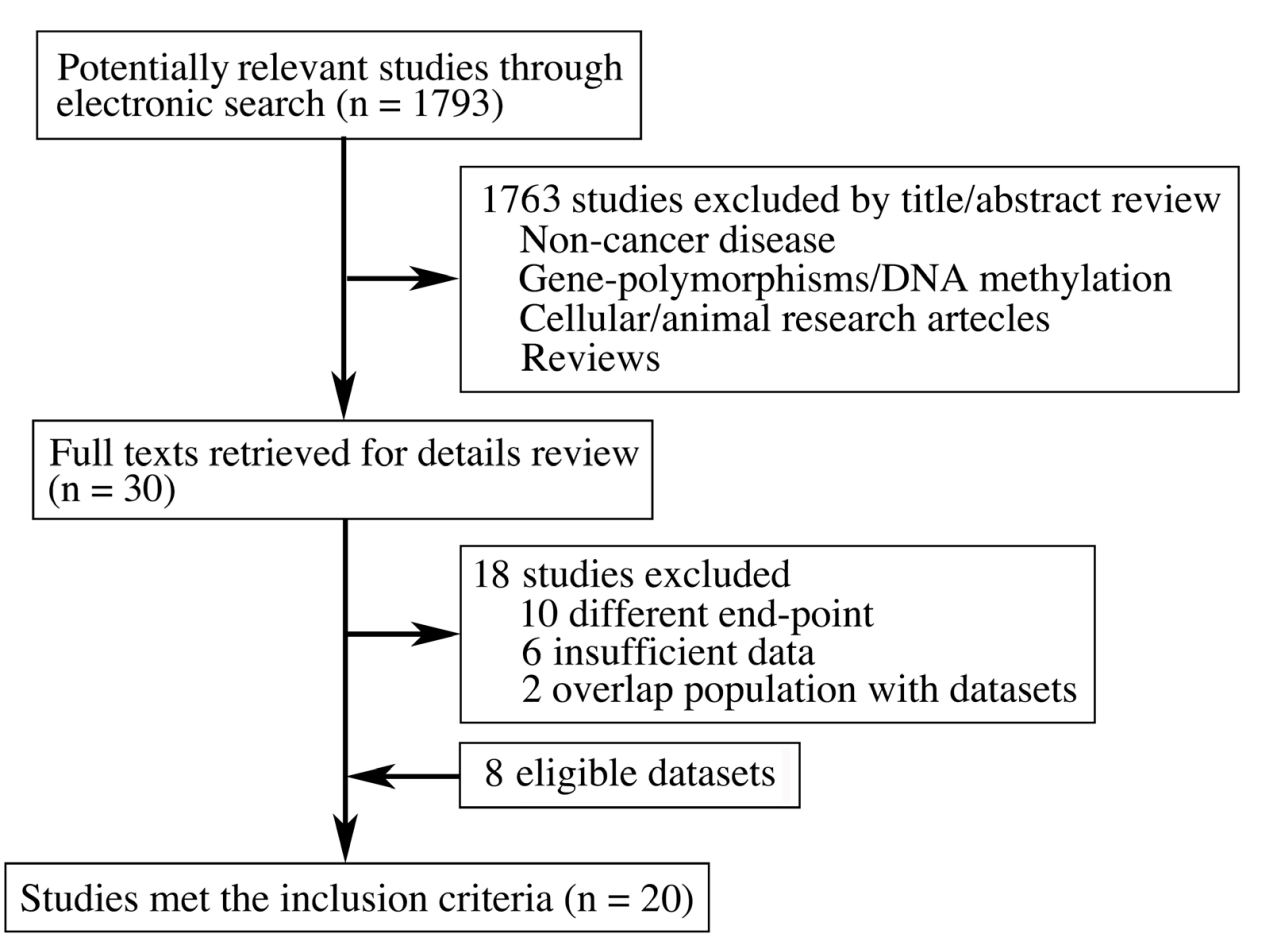
Flowchart of study selection

A total of 1793 literatures were identified from electronic search. Full texts of thirty publications were retrieved after reviewing titles and abstracts. After exclusion of 18 literatures (Table S1), finally, twelve studies [16–27] and eight datasets (GSE3149, GSE9891, GSE26712, GSE30161, GSE14764, GSE26193, GSE14814 and TCGA) [28–35] with 2149 cases met the inclusion criteria (Figure 2 and Table 1).

**Table 1:** The characteristics of the studies included in the meta-analysis

Author (Year)	Country	Type of transporter	Case/ Control	Data origin	Cancer type	End-points	Detection methods
Hsu (2015)	China	CTR1	40/40	Study	Ovarian cancer	PFS	IHC
Yang (2015)	China	CTR1 ATP7A ATP7B	32/22 20/34 15/39	Study	Lung cancer	OS, TR	IHC
TCGA (2015)	USA	CTR1 CTR2 ATP7A ATP7B	174/331 129/376 324/181 174/331	Dataset	Ovarian cancer	OS, PFS, TR	Gene array
Ogane (2013)	Japan	CTR1	30/17	Study	Endometrial cancer	OS, DFS	IHC
Katagiri (2013)	Japan	ATP7B	25/61	Study	Ovarian cancer	OS, PFS	IHC
Chen (2012)	China	CTR1 ATP7A ATP7B	37/17 26/28 40/14	Study	Lung cancer	OS, PFS, TR	IHC
Li (2012)	China	ATP7A	37/52	Study	Lung cancer	OS	IHC
LeeJK (2012)	USA	CTR1 CTR2 ATP7A ATP7B	15/43 45/13 45/13 18/40	Dataset	Ovarian cancer	OS, PFS, TR	Gene array
LeeYY (2011)	Korea	CTR1 CTR2	20/20 20/20	Study	Ovarian cancer	PFS, TR	qrt-PCR
Birrer (2011)	USA	CTR1 CTR2 ATP7A ATP7B	89/95 57/127 70/114 122/62	Dataset	Ovarian cancer	OS, PFS	Gene array
Mateescu (2011)	France	CTR1 CTR2 ATP7A ATP7B	48/45 68/25 49/44 26/67	Dataset	Ovarian cancer	OS, PFS, TR	Gene array
Tsao (2010)	Canada	CTR1 CTR2 ATP7A ATP7B	30/20 24/26 29/21 27/23	Dataset	Lung cancer	OS	Gene array
Martinez-Balibrea (2009)	Spain	ATP7B	16/34	Study	Colorectal cancer	PFS, TR	qrt-PCR, IHC
Denkert (2009)	Germany	CTR1 CTR2 ATP7A ATP7B	24/54 43/35 35/43 30/48	Dataset	Ovarian cancer	OS, PFS	Gene array
Tothill (2008)	Australia	CTR1 CTR2 ATP7A ATP7B	118/124 183/59 95/147 117/125	Dataset	Ovarian cancer	OS, PFS	Gene array
Aida (2005)	Japan	ATP7B	19/32	Study	Endometrial cancer	OS, DFS	IHC
Nevins (2005)	USA	CTR1 CTR2 ATP7A ATP7B	30/85 76/39 71/44 85/30	Dataset	Ovarian cancer	OS	Gene array
Nakayama (2004)	Japan	ATP7B	37/67	Study	Ovarian cancer	OS, TR	IHC
Samimi (2003)	USA	ATP7A	25/29	Study	Mixed cancers	OS	IHC
Nakayama (2002)	Japan	ATP7B	36/46	Study	Ovarian cancer	OS	qrt-PCR
							

IHC: Immunochemistry; qRT-PCR: Real-time polymerase chain reaction; OS: Overall survival; PFS: Progression-free survival; DFS: Disease-free survival; TR: Treatment response

### Copper transporters and survival

**Figure 3:**
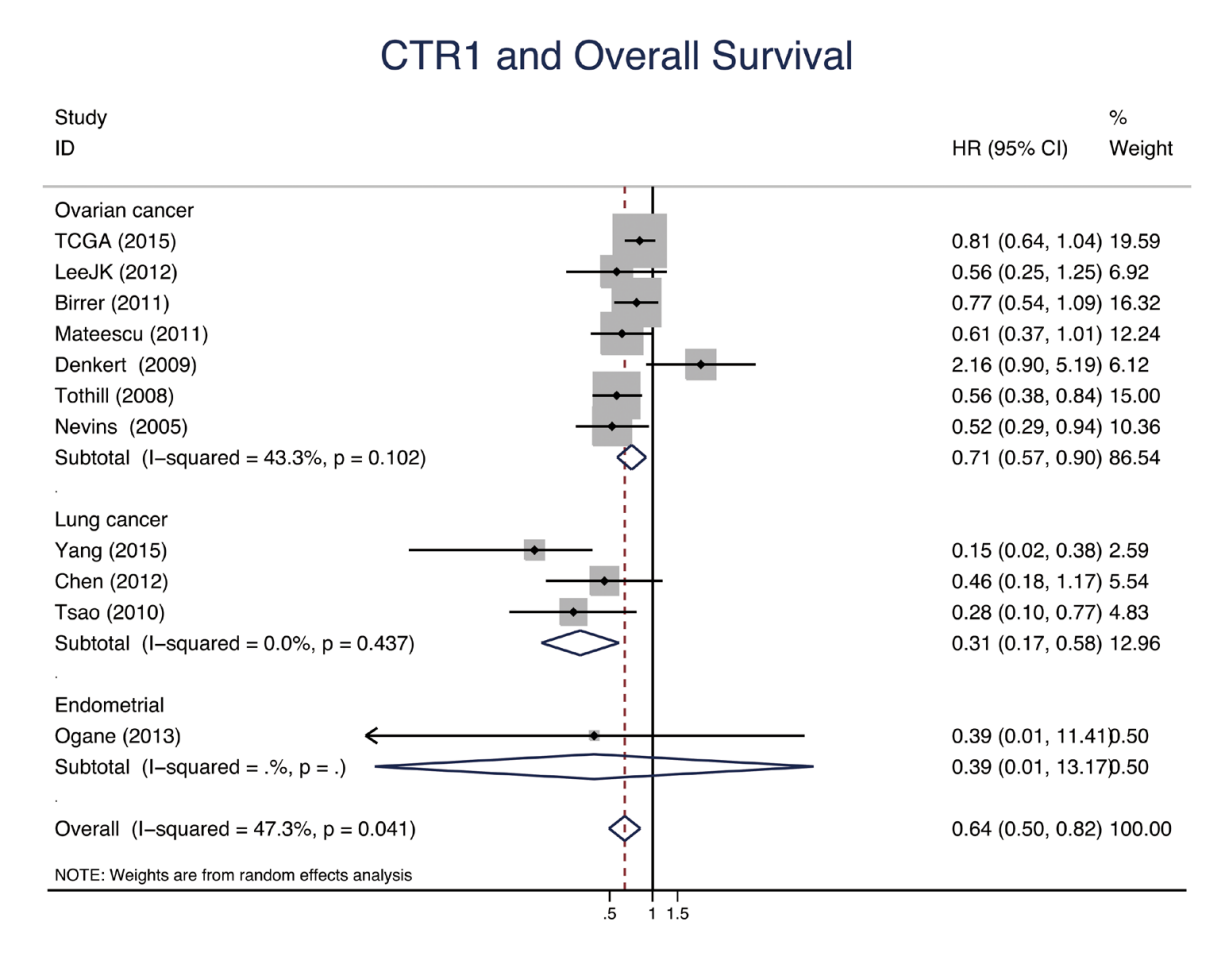
CTR1 and overall survival Forest plot presents summary hazard ratios (HRs) and 95% confidence intervals (CIs) of correlation between CTR1 expression and overall survival (OS) in epithelial cancers.

**Figure 4:**
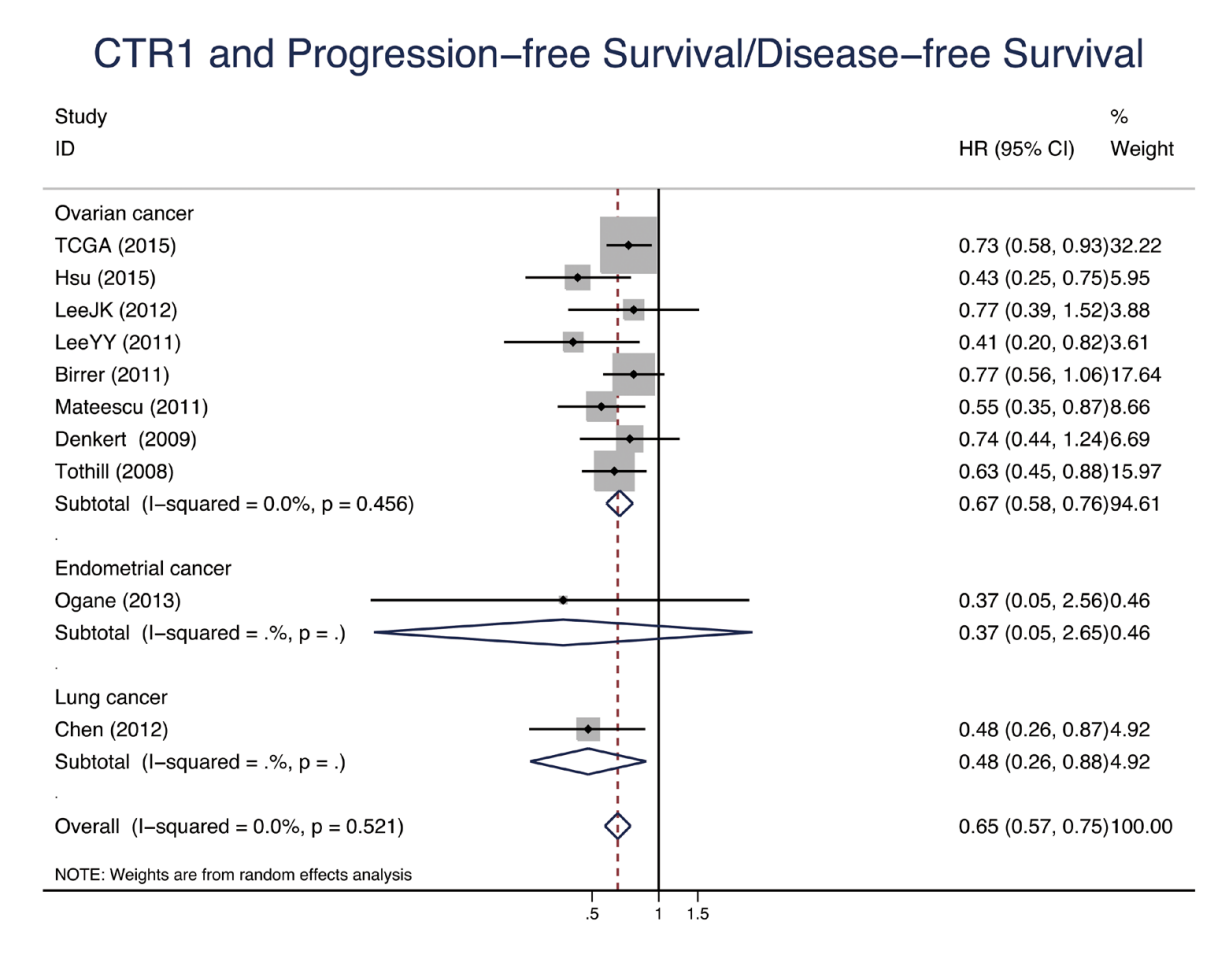
CTR1 and progression-free survival/disease-free survival Forest plot presents summary hazard ratios (HRs) and 95% confidence intervals (CIs) of correlation between CTR1 expression and progression free survival/disease-free survival (PFS/DFS in epithelial cancers.

**Figure 5:**
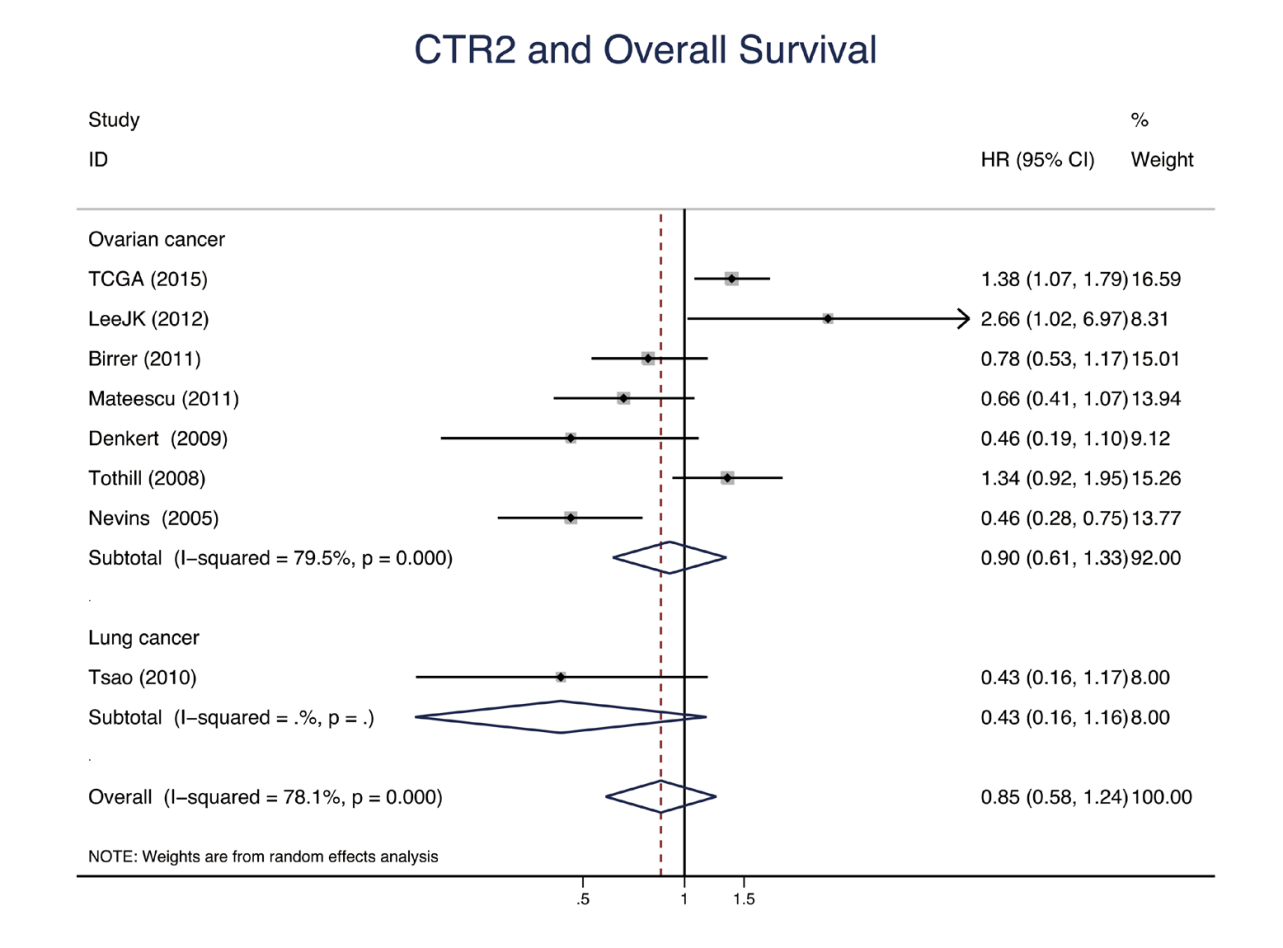
CTR2 and overall survival Forest plot presents summary hazard ratios (HRs) and 95% confidence intervals (CIs) of correlation between CTR2 expression and overall survival (OS) in epithelial cancers.

**Figure 6:**
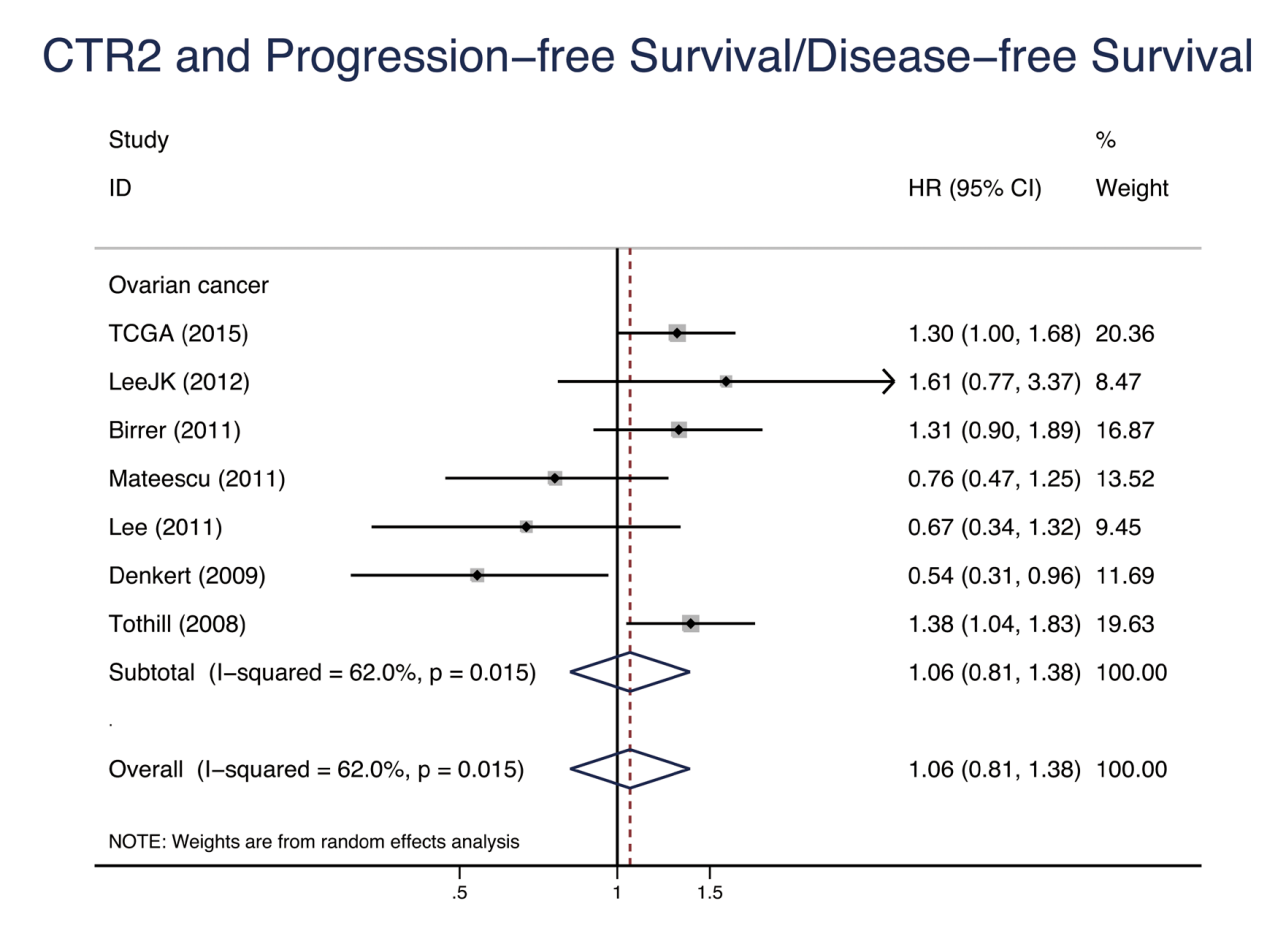
CTR2 progression-free survival/disease-free survival Forest plot presents summary hazard ratios (HRs) and 95% confidence intervals (CIs) of correlation between CTR2 expression and progression free survival/disease-free survival (PFS/DFS in epithelial cancers.

**Figure 7:**
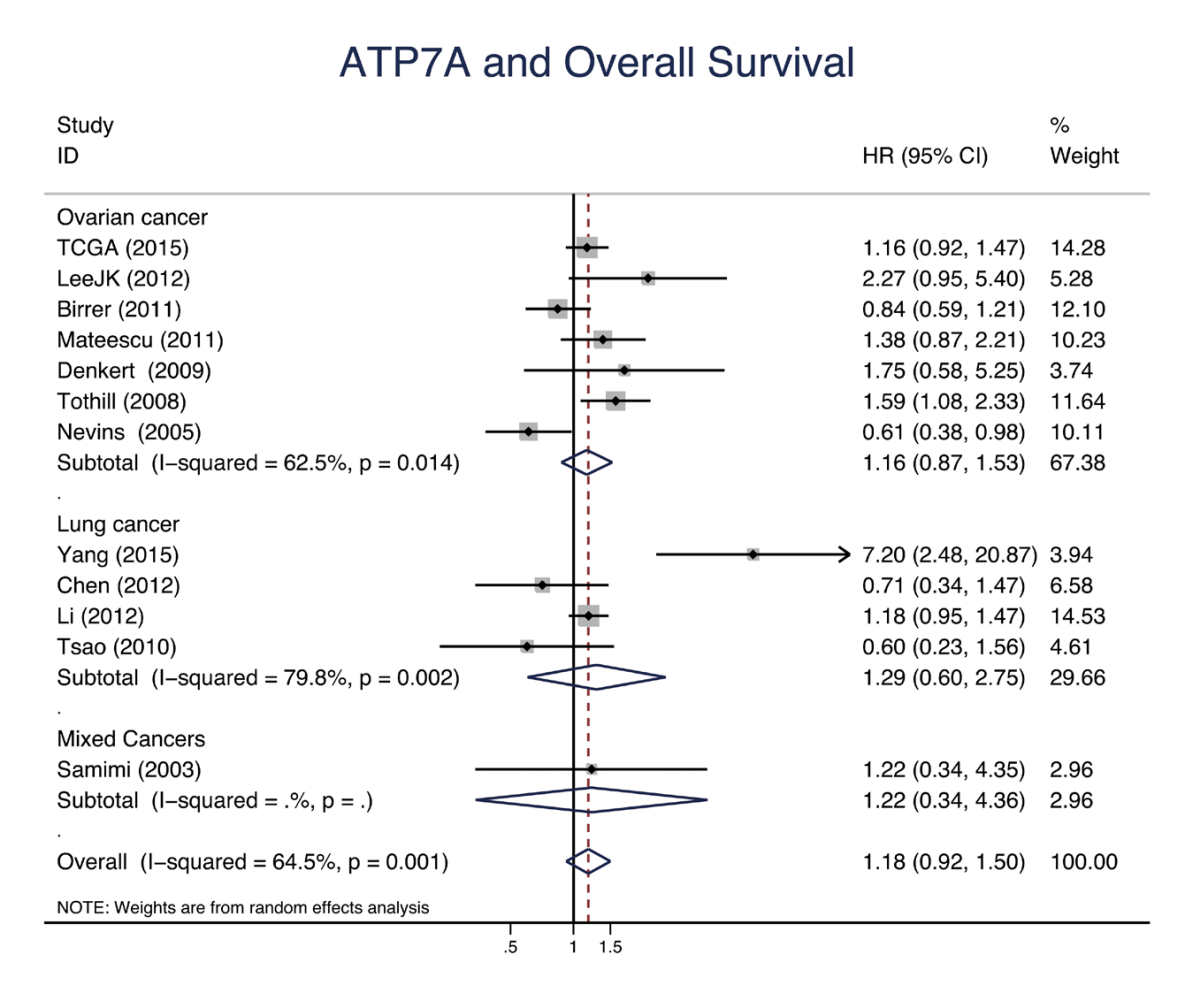
ATP7A and overall survival Forest plot presents summary hazard ratios (HRs) and 95% confidence intervals (CIs) of correlation between ATP7A expression and overall survival (OS) in epithelial cancers.

**Figure 8:**
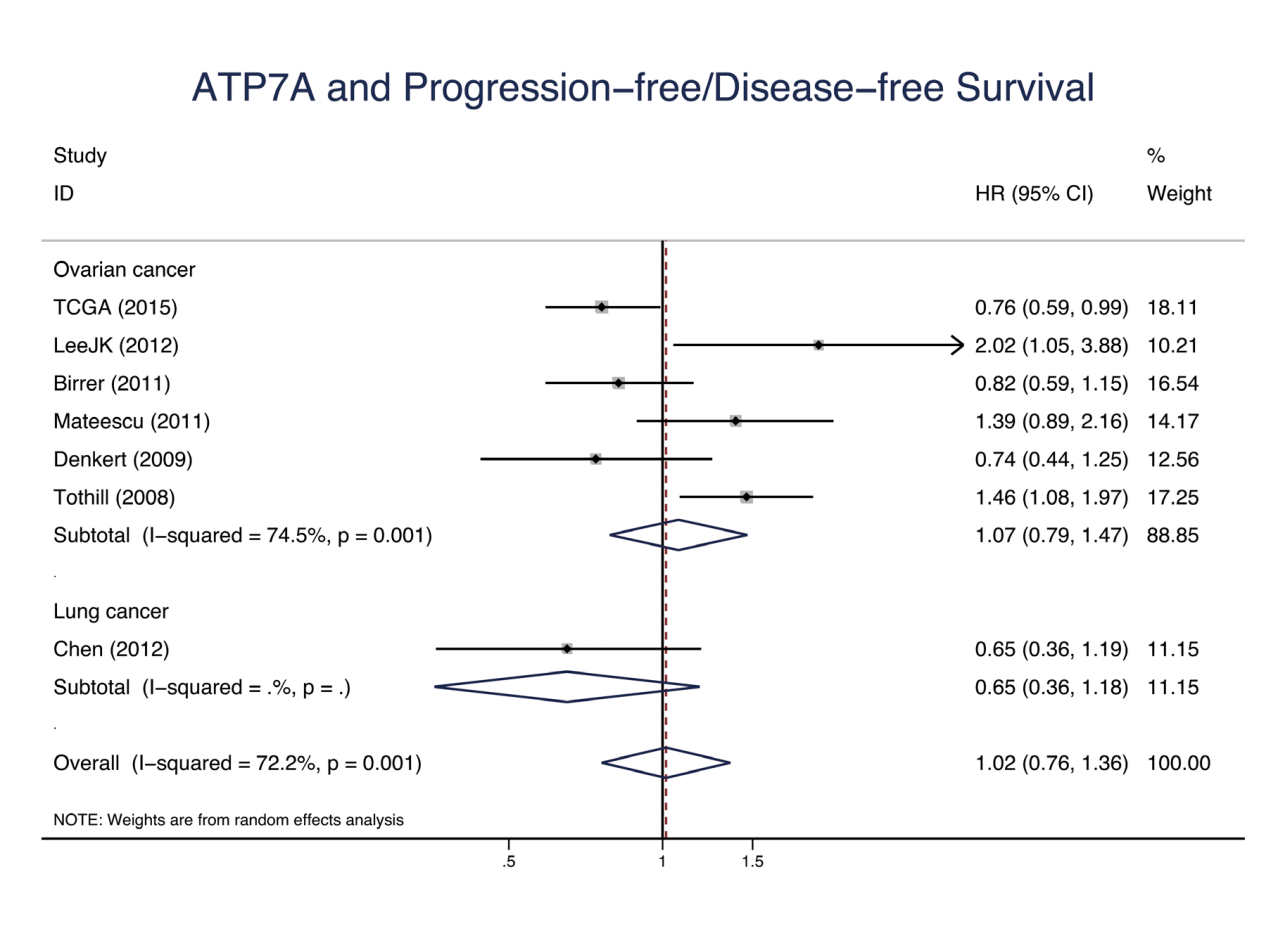
ATP7A and progression-free survival/disease-free survival Forest plot presents summary hazard ratios (HRs) and 95% confidence intervals (CIs) of correlation between ATP7A expression and progression free survival/disease-free survival (PFS/DFS in epithelial cancers.

**Figure 9:**
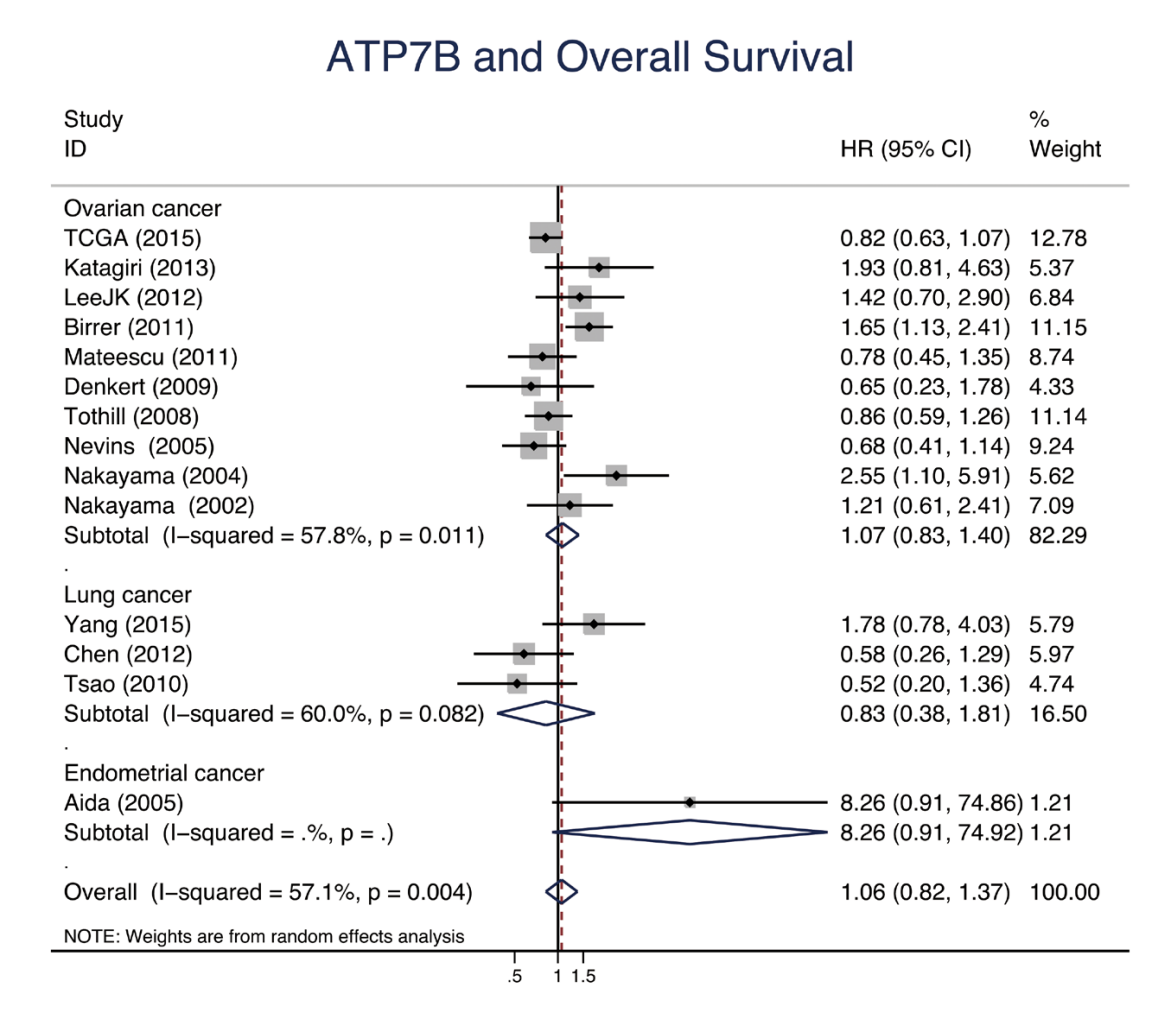
ATP7B and overall survival Forest plot presents summary hazard ratios (HRs) and 95% confidence intervals (CIs) of correlation between ATP7B expression and overall survival (OS) in epithelial cancers.

**Figure 10:**
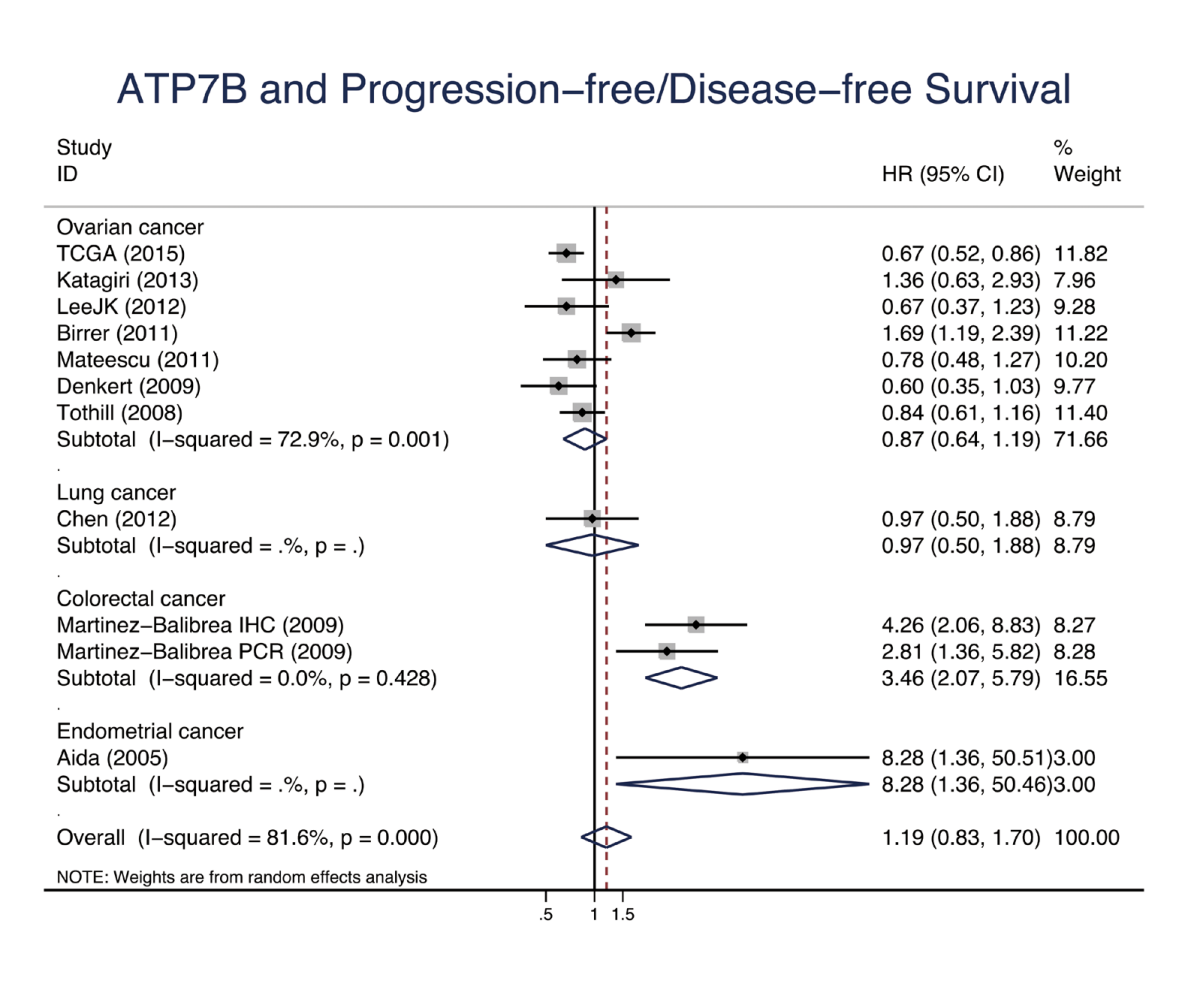
ATP7B and progression-free survival/disease-free survival Forest plot presents summary hazard ratios (HRs) and 95% confidence intervals (CIs) of correlation between ATP7B expression and progression free survival/disease-free survival (PFS/DFS in epithelial cancers.

Eleven studies evaluated the relationship between CTR1 expression and OS of cancer patients who received chemotherapy, and ten studies evaluated PFS/DFS. High CTR1 expression was correlated with a favorable OS and PFS in cancers (for OS, HR = 0.64, 95% CI: 0.50-0.82, *P* = 0.000; for PFS, HR = 0.65, 95% CI: 0.57-0.75, *P* = 0.000) (Figure 3 and Figure 4). The heterogeneities across studies included were moderate and acceptable (for OS, *I*^2^ = 47.3%; for PFS, *I*^2^ = 0.0%). However, there was no significant relation of the expression of CTR2, ATP7A and ATP7B to OS or PFS/DFS (Figure 5-10).

### Subgroup analysis of survival

Subgroup analysis was performed by stratifying data according to cancer type, chemotherapy regimen, geographic region, data origin and detection method. High CTR1 expression was significantly correlated with a favorable OS in the subgroups of “ovarian cancer” (HR = 0.71, 95% CI: 0.57-0.90, *P* = 0.004), lung cancer” (HR = 0.31, 95% CI: 0.17-0.58, *P* = 0.000), “platinum-based” (HR = 0.63, 95% CI: 0.49-0.81, *P* = 0.000), “America” (HR = 0.68, 95% CI: 0.82-0.88, *P* = 0.003), “Asia” (HR = 0.34, 95% CI: 0.16-0.73, *P* = 0.006), “dataset” (HR = 0.86, 95% CI: 0.53-0.87, *P* = 0.002), “literature” (HR = 0.34, 95% CI: 0.16-0.73, *P* = 0.006), “gene array” (HR = 0.86, 95% CI: 0.53-0.87, *P* = 0.002) and “IHC” (HR = 0.34, 95% CI: 0.16-0.73, *P* = 0.006). Similarly, high CTR1 expression was associated with an improved PFS/DFS in the subgroups of “ovarian cancer” (HR = 0.67, 95% CI: 0.58-0.76, *P* = 0.000), “platinum-based” (HR = 0.63, 95% CI: 0.54-0.73, *P* = 0.000), “America” (HR = 0.75, 95% CI: 0.62-0.90, *P* = 0.002), “Asia” (HR = 0.44, 95% CI: 0.31-0.62, *P* = 0.000), “Europe” (HR = 0.63, 95% CI: 0.44-0.88, *P* = 0.007), “dataset” (HR = 0.70, 95% CI: 0.61-0.81, *P* = 0.000), “literature” (HR = 0.44, 95% CI: 0.31-0.62, *P* = 0.000), “gene array” (HR = 0.70, 95% CI: 0.61-0.81, *P* = 0.000), “IHC” (HR = 0.47, 95% CI: 0.26-0.84, *P* = 0.010) and “PCR” (HR = 0.42, 95% CI: 0.27-0.65, *P* = 0.000) (Table 2). The heterogeneities were moderate (*I*^2^ range, 0.0% - 49.5%) and acceptable. High CTR2 was associated with an unfavorable PFS in the “America” subgroup (HR = 1.32, 95% CI: 1.08-1.62, *P* = 0.007), while in the “Europe” subgroup high CTR2 was associated with a favorable OS (HR = 0.61, 95% CI: 0.40-0.93, *P* = 0.020) and PFS/DFS (HR = 0.66, 95% CI: 0.45-0.95, *P* = 0.026). No evidence of a significant heterogeneity was found (Table 2). High ATP7B was correlated with a poor PFS in the “literature” subgroup (HR = 2.27, 95% CI: 1.16-4.42, *P* = 0.016) with a significant heterogeneity (*I*^2^ = 69.0) (Table 3).

**Table 2:** Subgroup analyses of CTR1 and CTR2 for survival

	End-point	CTR1					CTR2			
		N	HR (95%CI)	*P*	I^2^ (%)		N	HR (95%CI)	*P*	I^2^ (%)
Cancer type										
Ovarian cancer	OS	7	0.71(0.57, 0.90)	0.004	43.3		7	0.90(0.61, 1.33)	0.609	79.5
	PFS/DFS	8	0.67(0.58, 0.76)	0.000	0.0		7	1.06(0.81, 1.38)	0.679	62.0
Lung cancer	OS	3	0.31(0.17, 0.58)	0.000	0.0		1	0.43 (0.16, 1.16)	0.096	-
	PFS/DFS	1	0.48 (0.26, 0.88)	0.017	-		-	-	-	-
Endometrial cancer	OS	1	0.39 (0.01, 13.17)	0.600	-		-	-	-	-
	PFS/DFS	1	0.37 (0.05, 2.65)	0.322	-		-	-	-	-
Chemotherapy regimen										
Platinum-based	OS	11	0.63(0.49, 0.81)	0.000	47.1		8	0.79(0.55, 1.12)	0.179	72.6
	PFS/DFS	10	0.63(0.54, 0.73)	0.000	11.7		7	1.11(0.89, 1.38)	0.362	43.8
Geographic region										
America	OS	5	0.68(0.52, 0.88)	0.003	31.9		5	0.88(0.51, 1.54)	0.659	83.2
	PFS/DFS	3	0.75(0.62, 0.90)	0.002	0.0		3	1.32(1.08, 1.62)	0.007	0.0
Asia	OS	3	0.34(0.16, 0.73)	0.006	0.0		-	-	-	-
	PFS/DFS	4	0.44(0.31, 0.62)	0.000	0.0		1	0.67(0.34, 1.32)	0.247	-
Europe	OS	2	1.09(0.32, 3.74)	0.893	83.4		2	0.61(0.40, 0.93)	0.020	47.9
	PFS/DFS	2	0.63(0.44, 0.88)	0.007	0.0		2	0.66(0.45, 0.95)	0.026	0.0
Oceania	OS	1	0.49 (0.33, 0.73)	0.004	-		1	1.34(0.92, 1.95)	0.127	-
	PFS/DFS	1	0.51 (0.38, 0.69)	0.007	-		1	1.38(1.04, 1.83)	0.025	-
Data origin										
Dataset	OS	8	0.86(0.53, 0.87)	0.002	49.5		8	0.85(0.58, 1.24)	0.403	78.1
	PFS/DFS	6	0.70(0.61, 0.81)	0.000	0.0		6	1.11(0.85, 1.45)	0.437	61.9
Literature	OS	3	0.34(0.16, 0.73)	0.006	0.0		-	-	-	-
	PFS/DFS	4	0.44(0.31, 0.62)	0.000	0.0		1	0.67(0.34, 1.32)	0.247	-
Detection method										
Gene array	OS	8	0.86(0.53, 0.87)	0.002	49.5		8	0.85(0.58, 1.24)	0.403	78.1
	PFS/DFS	6	0.70(0.61, 0.81)	0.000	0.0		6	1.11(0.85, 1.45)	0.437	61.9
IHC	OS	3	0.34(0.16, 0.73)		0.0		-	-	-	-
	PFS/DFS	2	0.47 (0.26, 0.84)	0.010	0.0		-	-	-	-
qRT-PCR	OS	-	-	-	-		-	-	-	-
	PFS/DFS	2	0.42 (0.27, 0.65)	0.000	0.0		1	0.67 (0.34, 1.32)	0.247	-
										

**Table 3:** Subgroup analyses of ATP7A and ATP7B for survival

	End-point	ATP7A					ATP7B			
		N	HR (95%CI)	P	I^2^ (%)		N	HR (95%CI)	P %	I^2^ (%)
Cancer type										
Ovarian cancer	OS	7	1.16(0.87, 1.53)	0.306	62.5		10	1.07(0.83, 1.40)	0.594	57.8
	PFS/DFS	6	1.07(0.79, 1.47)	0.651	74.5		7	0.87(0.64, 1.19)	0.387	72.9
Lung cancer	OS	4	1.29(0.60, 2.75)	0.514	79.8		3	0.83(0.38, 1.81)	0.633	60.0
	PFS/DFS	1	0.65(0.36, 1.18)	0.158	-		1	0.97(0.50, 1.88)	0.928	-
Endometrial cancer	OS	-	-	-	-		1	8.26(0.91, 74.92)	0.061	-
	PFS/DFS	-	-	-	-		1	3.46(2.07, 5.79)	0.022	-
Colorectal cancer	OS	-	-	-	-		-	-	-	-
	PFS/DFS	-	-	-	-		1	8.28(1.36, 50.46)	0.000	-
Mixed cancers	OS	1	1.22(0.34, 4.36)	0.188	-		-	-	-	-
	PFS/DFS	-	-	-	-		-	-	-	-
Chemotherapy regimen										
Platinum-based	OS	12	1.16(0.92, 1.48)	0.170	62.2		14	1.06(0.82, 1.37)	0.650	57.1
	PFS/DFS	7	1.04(0.77, 1.39)	0.754	72.1		11	1.23(0.86, 1.75)	0.255	81.4
Geographic region										
America	OS	6	0.95(0.69, 1.32)	0.762	55.5		5	0.97(0.65, 1.45)	0.882	70.8
	PFS/DFS	3	0.97(0.64, 1.49)	0.902	73.3		3	0.92(0.48, 1.79)	0.812	89.3
Asia	OS	3	1.63(0.63, 4.18)	0.313	84.4		6	1.54(0.92, 2.59)	0.103	50.4
	PFS/DFS	1	0.65(0.36, 1.18)	0.158	-		3	1.56(0.67, 3.63)	0.298	58.3
Europe	OS	2	1.43(0.93, 2.20)	0.102	0.0		2	0.75(0.46, 1.21)	0.241	0.0
	PFS/DFS	2	1.03(0.56, 1.91)	0.925	69.3		3	1.49(0.61, 3.65)	0384	88.6
Oceania	OS	1	1.59(1.08, 2.34)	0.018	-		1	0.86(0.59, 1.26)	0.436	-
	PFS/DFS	1	1.46(1.08, 1.97)	0.014	-		1	0.84(0.61, 1.16)	0.288	-
Data origin										
Dataset	OS	8	1.11(0.85, 1.46)	0.444	60.3		8	0.91(0.70, 1.18)	0.491	53.1
	PFS/DFS	6	1.07(0.79, 1.47)	0.651	74.5		6	0.83(0.60, 1.16)	0.278	75.8
Literature	OS	4	1.51(0.71, 3.24)	0.285	76.7		6	1.54(0.92, 2.59)	0.103	50.4
	PFS/DFS	1	0.65(0.36, 1.18)	0.158	-		5	2.27(1.16, 4.42)	0.016	69.0
Detection method										
Gene array	OS	8	1.11(0.85, 1.46)	0.444	60.3		8	0.91(0.70, 1.18)	0.491	53.1
	PFS/DFS	6	1.07(0.79, 1.47)	0.651	74.5		6	0.83(0.60, 1.16)	0.278	75.8
IHC	OS	4	1.51(0.71, 3.24)	0.285	76.7		5	1.69(0.86, 3.30)	0.126	58.8
	PFS/DFS	1	0.65(0.36, 1.18)	0.158	-		4	2.19(0.92, 5.22)	0.075	74.9
qRT-PCR	OS	-	-	-	-		1	1.21(0.61, 2.41)	0.587	-
	PFS/DFS	-	-	-	-		1	2.81(1.36, 5.81)	0.005	-
										

P-value for significance of HR. (P < 0.050) HR: hazard ratio; CI: confidence interval ; IHC: immunochemistry; qRT-PCR: real-time polymerase chain reaction

*P*-value for significance of OR. (P < 0.050) HR: hazard ratio; CI: confidence interval ; IHC: immunochemistry; qRT-PCR: real-time polymerase chain reaction

### Copper transporters and treatment response

Six studies evaluated CTR1 expression and TR of cancer patients who received adjuvant chemotherapy. High CTR1 expression was associated with increased TR (OR = 2.99, 95% CI: 1.57-5.69, *P* = 0.001), especially in the subgroups of “ovarian cancer” (OR = 2.28, 95% CI: 1.15-4.54, *P* = 0.019), “lung cancer” (OR = 5.64, 95% CI: 2.17-14.65, *P* = 0.000), “Asia” (OR = 6.39, 95% CI: 2.83-14.41, *P* = 0.000), “dataset” (OR = 1.61, 95% CI: 1.10-2.37, *P* = 0.015), “literature” (OR = 6.39, 95% CI: 2.83-14.41, *P* = 0.000), “gene array” (OR = 1.61, 95% CI: 1.10-2.37, *P* = 0.015) and “IHC” (OR = 5.64, 95% CI: 2.17-14.65, *P* = 0.000) subgroups. The heterogeneities were moderate and acceptable. No evidence for a correlation between CTR2, ATP7A or ATP7B expression and TR was found (Table 4).

**Table 4:** Meta-analysis and subgroups analyses of treatment response

	CTR1					CTR2			
	N	OR (95%CI)	*P*	I^2^ (%)		N	OR (95%CI)	*P*	I^2^ (%)
All studies	6	2.99(1.57, 5.69)	0.001	52.6		4	0.82(0.55, 1.21)	0.308	0.0
Cancer type									
Ovarian cancer	4	2.28(1.15, 4.54)	0.019	47.1		4	0.82(0.55, 1.21)	0.308	0.0
Lung cancer	2	5.64(2.17, 14.65)	0.000	0.0		-	-		-
Geographic region									
America	2	1.49(0.99, 2.25)	0.056	0.0		2	0.86(0.54, 1.38)	0.542	0.0
Asia	3	6.39(2.83, 14.41)	0.000	38.74		1	0.41(0.11, 1.56)	0.190	-
Europe	1	2.93(0.94, 9.13)	0.063	-		1	0.89(0.40, 2.00)	0.778	-
Data origin									
Dataset	3	1.61(1.10, 2.37)	0.015	0.0		3	0.87(0.58, 1.31)	0.503	0.0
Literature	3	6.39(2.83, 14.41)	0.000	0.0		1	0.41(0.11, 1.56)	0.190	
Detection method									
Gene array	3	1.61(1.10, 2.37)	0.015	0.0		3	0.87(0.58, 1.31)	0.503	0.0
IHC	2	5.64(2.17, 14.65)	0.000	0.0		-	-	-	-
qRT-PCR	1	8.90(1.89, 41.98)	0.006	-		1	0.41(0.11, 1.56)	0.190	-
									

	ATP7A					ATP7B			
	N	HR (95%CI)	*P*	I^2^ (%)		N	HR (95%CI)	*P*	I^2^ (%)
All studies	7	0.94(0.59, 1.52)	0.808	47.7		7	0.91(0.65, 1.27)	0.571	5.8
Cancer type									
Ovarian cancer	3	0.95(0.64, 1.41)	0.795	0		4	0.82(0.49, 1.37)	0.443	40.4
Lung cancer	3	0.61(0.18, 2.09)	0.431	72.9		2	1.60(0.66, 3.90)	0.299	0.0
Colorectal cancer	1	1.94(0.76, 4.95)	0.164	-		1	0.71(0.29, 1.78)	0.469	-
Geographic region									
America	2	0.80(0.51, 1.27)	0.353	0		2	1.12(0.71, 1.77)	0.633	0.0
Asia	3	0.61(0.18, 2.09)	0.431	72.9		3	0.91(0.30, 2.77)	0.863	0.0
Europe	2	1.66(0.92, 3.01)	0.092	0		2	0.74(0.40, 1.37)	0.338	0.0
Data origin									
Dataset	3	0.95(0.64, 1.41)	0.886	0		3	1.02(0.68, 1.53)	0.908	0.0
Literature	4	0.94(0.38, 2.30)	0.795	65.1		4	0.79(0.42, 1.50)	0.477	31.1
Detection method									
Gene array	4	0.80(0.28, 2.27)	0.795	69.4		3	1.02(0.68, 1.53)	0.908	0.0
IHC	3	0.95(0.64, 1.41)	0.668	0		4	0.84(0.37, 1.88)	0.664	48.3
qRT-PCR	1	1.94(0.52, 7.29)	0.325	-		1	0.71(0.20, 2.59)	0.609	-
									

P-value for significance of HR. (P < 0.050) HR: hazard ratio; CI: confidence interval ; IHC: immunochemistry; qRT-PCR: real-time polymerase chain reaction

### Sensitivity analysis and Publication bias

According to sensitivity analyses, the summary HRs for OS of CTR1 and PFS of CTR2 were influenced by one study in the “Europe” subgroup [32]. All the remaining studies did not influence the summary HRs and ORs. No evidence of publication bias was found in overall meta-analyses using funnel plots and Egger's test (all *P* > 0.05).

## DISCUSSION

In this meta-analysis, we systematically evaluated the relationship between four copper transporters (CTR1, CTR2, ATP7A and ATP7B) that influence cellular platinum accumulation and prognosis of cancer patients who received chemotherapy. Our results showed that high CTR1 expression was significantly associated with a favorable OS, PFS, DFS and TR, suggesting that CTR1 is a potential prognostic factor for survival in cancer patients underwent chemotherapy and a treatment target for overcoming platinum resistance.

Ever since the introduction of chemotherapy, drug response becomes a major determiner of cancer prognosis, which parallels with FIGO stage and residual debulked tumor volume. The cure rate of testicular cancer zoomed from 15% to 90% attributing to a great response to platinum-based chemotherapy[36]. In ovarian cancer patients, different histologic subtypes are characterized by distinct platinum response leading to different prognosis, the better the response the better the survival. Switching on and off of an array of genes such as BRCA1, BRCA2, Akt1, ERCC1 and ABCB1 were identified to modulate sensitivity toward chemotherapy in cancer patients. We demonstrated that CTR1 is one of those genes whose expression is significantly associated with OS, PFS, DFS and chemotherapy response in patients underwent chemotherapy, especially in those who received platinum-based chemotherapy. CTR1 contributes to the active cellular uptake of cisplatin, carboplatin and oxaliplatin. In our study, most patients received cisplatin- or carboplatin-based chemotherapy, both of which are important substrates of hCTR1. It was reported that hCTR1 was responsible to a 60% to 70% cellular uptake of cisplatin and a 30% to 40% uptake of carboplatin [37, 38]. So far, no other chemotherapy agents were identified as CTR1 substrates. Unfortunately, we were not able to analyze the impact of CTR1 on patients underwent non-platinum containing chemotherapy due to insufficient data. We found a significant association between CTR1 expression and tumor prognosis by pooling data from 5 studies and 8 datasets involving 2149 patients. Although all data were retrospectively obtained, multiple data sources greatly decreased the publication bias and efficiently increased the validity of the results. Significant clinical associations of CTR1 expression were constant between all subgroups: cancer type, geographical region, data origin and detection method with acceptable heterogeneities.

In our study, we focused on evaluating the initial copper transporter expression in blood and tissue specimens obtained before or during primary surgery. We found that a high initial hCTR1 expression predicted improved prognosis in ovarian and lung cancer patients. However, pre-clinical evidence indicates that the expression of CTR1 is dynamic. Prompt reaction of CTR1 to acute copper concentration alteration is a well-recognized mechanism that regulates hCTR1 expression. Excessive copper triggers rapid hCTR1 internalization by endocytosis, which reduces the transportation activity of CTR1 [39, 40]. Copper deficiency up-regulates transcription-factor-specific protein 1 (Sp1), which binds to hCTR1 promoter region and enhances hCTR1 expression[41]. Some studies also reported that cisplatin induces rapid hCTR1 degradation through ubiquitin-mediated proteolysis [42, 43]. According to Jandial et al., administration of proteasome inhibitor bortezomib successfully blocked platinum-induced CTR1 degradation and increased platinum accumulation in peritoneal tumors [44]. Whether hCTR1 expression is subjected to similar dynamic change induced by substrate stimulation in cancer patients and whether this change is associated with tumor prognosis are still undetermined.

In relation to chemotherapy response and cancer prognosis, according to our results, CTR1 is of particular attractiveness to serve as a promising target to circumvent platinum resistance. Since correlation between reduced hCTR1 expression and platinum resistance was exhibited in precedent and the present studies, combination of copper lowering agents and platinum based chemotherapy, which stimulates hCTR1 expression and improve platinum uptake, is a promising strategy to treat hCTR1 deficiency mediated platinum resistance. Ishida S et al. successfully enhanced the cytotoxic effect of cisplatin on human cervical and ovarian cancer cells by up-regulating hCTR1 expression through administration of a copper chelating agent tetrathiomolybdate in vitro [45]. Fu et al. carried out the first clinical trial to explore the clinical value of copper chelating agents. Approximate 20% of the platinum resistant patients responded to platinum after receiving copper chelator [46]. However, this strategy was not tested in clinical trials.

We initially expected diverse prognostic values of CTR2 in distinct cancer types, in patients with different genetic background and in studies applied inconsistent CTR2 detection methods based on existing studies. Though CTR2 shares a substantial similar structure with CTR1, it plays drastically different role in platinum uptake. Unlike CTR1, preclinical studies revealed that high CTR2 level was associated with decreased cellular platinum uptake and poor drug response in ovarian cancer [8, 47]. Additionally, copper modulation correlated CTR2 with angiogenesis in clear cell renal cell carcinoma, where lower CTR2 expression predicted shorter OS and DFS [48]. Heterogeneity of genetic background of patients not only influences basic CTR2 level of the population but also alters various gene levels and pathways that CTR2 interacts with. Moreover, CTR2 regulates cellular platinum uptake through a protein-protein interaction with CTR1 [8], the relationship between CTR2 and platinum sensitivity is better revealed post-transcriptionally. When evaluating the relationship of CTR2 and cancer prognosis in a clinical setting, we did not find any evidence for correlation between CTR2 and prognosis in different cancer types, data origins and detection methods but saw a discrepancy in geographic stratification of the America and the Europe subgroups regarding PFS. We found that the detection method, cancer type, methodology, type of samples were consistent in all 5 studies from these two subgroups. One study from the Europe subgroup reported a specific result that was opposite to all other studies [32]. We did not have enough evidence to determine whether the inconsistent result was due to genetic difference or simply small size effect. We believed it was necessary to investigate the issue in a much greater clinical scale and covered as much as detection methods as possible since our results were only obtained on the basis of a series gene-array analyses due to limited data, which might not necessarily exhibit the correlation between CTR2 protein expression and platinum resistance.

The present study is the first meta-analysis regarding the impact of copper transporters on the outcomes of cancer patients who received chemotherapy to our knowledge. We used multiple data sources and performed subgroup analyses stratified by geological regions of publication, data origins and detection methods to accommodate possible biases and provide detailed results that help accelerate the relevant clinical translation. However, all the enrolled studies are retrospective, which provide evidence of Level II-1 or II-2 [49] and limit the reliability of our findings. The relationship between CTR1 and chemotherapy clinical outcomes in tumor patients needs validation by large-scale randomized controlled trials. In addition, most data for meta-analysis were obtained from studies on ovarian cancer and lung cancer, which restricts the application of the conclusions in other malignancies.

To sum up, high CTR1 level is associated with improved survival and TR in cancer patients, especially in patients with ovarian or lung cancer undergoing platinum-based chemotherapy, Prospective randomized studies are needed to verify the clinical value of CTR1in tumor patients.

## MATERIALS AND METHODS

### Search strategy

An independent systematic literature search of studies in all languages to May 2016 across PubMed, EMBASE, and Chinese Wanfang databases was conducted. The search terms used were “copper transporter 1”, “human copper transporter 1”, “copper transporter 2”, “ATP7A”, “ATP7B” and “platinum-based chemotherapy”. The electronic search of literature was supplemented by reviewing the reference lists of retrieved studies. Additionally we searched the Gene Expression Omnibus (GEO) (http://www.ncbi.nlm.nih.gov/gds) and the Cancer Genome Atlas (TCGA) datasets (http://cancergenome.nih.gov) for relevant information. The key words used were “CTR1”, “CTR2”, “ATP7A”, “ATP7B”, “platinum” and “Homo sapiens”.

### Study Selection

A two-step study selection was conducted including initial review of titles and abstracts, and a second review of full texts retrieved. The inclusion criteria for identifying eligible studies included: 1) clinical studies measured CTR1, CTR2, ATP7A or ATP7B in solid tumors regardless of detection methods; 2) published prospective and retrospective cohort studies; 3) studies provided information of survival and chemotherapy response. Exclusion criteria included: 1) the patients enrolled did not receive chemotherapy; 2) studies focused on gene polymorphisms or DNA methylation; 3) studies investigated different end-points; 4) studies did not provide sufficient data. Eligible datasets should meet the following criteria: 1) cancer patients received chemotherapy; 2) the sample size should be more than 40; 3) gene array was performed using Affymetrix platform. If multiple studies shared overlapped populations, the study with the largest sample size was included. If multiple studies with overlapped populations used different detection methods, all were included.

### Quality assessment and Data extraction

The quality of the included literatures was assessed according to the Newcastle-Ottawa Scale (NOS) for cohort studies[50]. We extracted the following data from each individual eligible study or dataset: first author’s/contributor's name, year of publication, country, sample size, number of cases and controls, tumor type, detection methods and detection results. Two authors extracted the data independently and disagreements were solved by discussion.

### Statistical Methods

The aim of our study was to investigate the relationship between the expression of copper transporters and the prognosis of cancer patients who received chemotherapy. The primary outcome measure was overall survival (OS), and secondary measures were progression-free survival (PFS)/disease-free survival (DFS) and treatment response (TR). The impact of platinum-transporter expression on OS, PFS and DFS was assessed by hazard ratio (HR), odds ratio (OR) in case of TR, and its 95% confidence interval (CI). The HRs and 95% CIs were extracted from eligible studies directly or calculated using the survival data extracted from the reported Kaplan-Meier curves with the software GetDATA Graph Digitizer 2.25 according to the method described by Tierney et al. [51]. An on-line survival analysis software KM-plotter (http://kmplot.com/analysis/) with the selection of optimal cut-off point and microarray probe set was used to calculate the HRs and 95% CIs using data from GEO and TCGA datasets [52–54]. Random-effects models were employed to pool the HRs, ORs and 95%CIs [55]. The heterogeneity across studies was assessed according to *I*2 statistic with the method of Higgins et al. [56]. A heterogeneity was considered significant when *I*2 ≥ 50%. Subgroup analyses stratified by tumor type, geographic region, data origin and detection method were performed. Sensitivity analyses were performed by excluding one study at a time to evaluate the influence of single studies on summary HRs. Publication bias was assessed by funnel plot and Egger's test [57]. All analyses were conducted using STATA14 (MP-Parallel Edition, College Station, Texas 77845 USA). *P* values less than 0.05 were considered statistically significant.

## SUPPLEMENTARY MATERIALS TABLE



## Abbreviations

HR: hazard ratio
CI: confidence interval
CTR1: copper transporter 1
hCTR1: human copper transporter 1
CTR2: copper transporter 2
ATP7A: Copper-transporting P-type adenosine triphosphatase 1
ATP7B: copper-transporting P-type adenosine triphosphatase 2
