# Supplementary material for: The association between copper transporters and the prognosis of cancer patients undergoing chemotherapy: a meta-analysis of literatures and datasets
Source: Oncotarget. 2016 Dec 12;8(9):16036–51. doi: 10.18632/oncotarget.13917 (PMC5362544; doi:10.18632/oncotarget.13917)
Supplement: Supplementary file 1 [file oncotarget-08-16036-s001.docx]

The association between copper transporters and the prognosis of cancer patients undergoing chemotherapy: a meta-analysis of literatures and datasets

**Supplementary Material**

**Table S1. The list of studies excluded and reasons for exclusion.**

| **Study** | **Reason for exclusion** |
| --- | --- |
| 1. Le Roy, B., Tixier, L., Pereira, B., Sauvanet, P., Buc, E., Petorin, C., Dechelotte, P., Pezet, D., and Balayssac, D. (2016) Assessment of the Relation between the Expression of Oxaliplatin Transporters in Colorectal Cancer and Response to FOLFOX-4 Adjuvant Chemotherapy: A Case Control Study. *PloS one* **11**, e0148739 | Insufficient data |
| 2. Kilari, D., Iczkowski, K. A., Pandya, C., Robin, A. J., Messing, E. M., Guancial, E., and Kim, E. S. (2016) Copper Transporter-CTR1 Expression and Pathological Outcomes in Platinum-treated Muscle-invasive Bladder Cancer Patients. *Anticancer research* **36**, 495-501 | Insufficient data |
| 3. Xia, Y., Liu, L., Long, Q., Bai, Q., Wang, J., Xu, J., and Guo, J. (2016) Decreased expression of CTR2 predicts poor prognosis of patients with clear cell renal cell carcinoma. Urologic oncology 34, 5 e1-9 | Insufficient data |
| 4. Wang, X., Jiang, P., Wang, P., Yang, C. S., Wang, X., and Feng, Q. (2015) EGCG Enhances Cisplatin Sensitivity by Regulating Expression of the Copper and Cisplatin Influx Transporter CTR1 in Ovary Cancer. *PloS one* **10**, e0125402 | Different end-point |
| 5. Tsimberidou, A. M., Said, R., Culotta, K., Wistuba, I., Jelinek, J., Fu, S., Falchook, G., Naing, A., Piha-Paul, S., Zinner, R., Siddik, Z. H., He, G., Hess, K., Stewart, D. J., Kurzrock, R., and Issa, J. P. (2015) Phase I study of azacitidine and oxaliplatin in patients with advanced cancers that have relapsed or are refractory to any platinum therapy. *Clinical epigenetics* **7**, 29 | Different end-point |
| 6. Takeda, R., Naka, A., Ogane, N., Kameda, Y., Kawachi, K., Shimizu, S., and Kamoshida, S. (2015) Impact of Expression Levels of Platinum-uptake Transporters Copper Transporter 1 and Organic Cation Transporter 2 on Resistance to Anthracycline/Taxane-based Chemotherapy in Triple-negative Breast Cancer. *Breast cancer : basic and clinical research* **9**, 49-57 | Different end-point |
| 7. Yoshida, H., Teramae, M., Yamauchi, M., Fukuda, T., Yasui, T., Sumi, T., Honda, K., and Ishiko, O. (2013) Association of copper transporter expression with platinum resistance in epithelial ovarian cancer. *Anticancer research* **33**, 1409-1414 | Insufficient data |
| 8. Schmid, S. C., Schuster, T., Horn, T., Gschwend, J., Treiber, U., and Weirich, G. (2013) Utility of ATP7B in prediction of response to platinum-based chemotherapy in urothelial bladder cancer. *Anticancer research* **33**, 3731-3737 | Different end-point |
| 9. Karachaliou, N., Papadaki, C., Lagoudaki, E., Trypaki, M., Sfakianaki, M., Koutsopoulos, A., Mavroudis, D., Stathopoulos, E., Georgoulias, V., and Souglakos, J. (2013) Predictive value of BRCA1, ERCC1, ATP7B, PKM2, TOPOI, TOPOmicron-IIA, TOPOIIB and C-MYC genes in patients with small cell lung cancer (SCLC) who received first line therapy with cisplatin and etoposide. *PloS one* **8**, e74611 | Insufficient data |
| 10. Kim, H., Kim, K., No, J. H., Jeon, Y. T., Jeon, H. W., and Kim, Y. B. (2012) Prognostic value of biomarkers related to drug resistance in patients with advanced epithelial ovarian cancer. Anticancer Res 32, 589-594 | Insufficient data |
| 11. Xu, X., Ren, H., Zhou, B., Zhao, Y., Yuan, R., Ma, R., Zhou, H., and Liu, Z. (2012) Prediction of copper transport protein 1 (CTR1) genotype on severe cisplatin induced toxicity in non-small cell lung cancer (NSCLC) patients. *Lung cancer* **77**, 438-442 | Different end-point |
| 12. Xu, X., Duan, L., Zhou, B., Ma, R., Zhou,H., and Liu, Z. (2012) Genetic polymorphism of copper transporter protein 1 is related to platinum resistance in Chinese non-small cell lung carcinoma patients. Clinical and experimental pharmacology & physiology 39, 786-792 | Different end-point |
| 13. Liang, Z. D., Long, Y., Tsai, W. B., Fu, S., Kurzrock, R., Gagea-Iurascu, M., Zhang, F., Chen, H. H., Hennessy, B. T., Mills, G. B., Savaraj, N., and Kuo, M. T. (2012) Mechanistic basis for overcoming platinum resistance using copper chelating agents. *Molecular cancer therapeutics* **11**, 2483-2494 | Overlap population with datasets |
| 14. Ishida, S., McCormick, F., Smith-McCune, K., and Hanahan, D. (2010) Enhancing tumor-specific uptake of the anticancer drug cisplatin with a copper chelator. *Cancer cell* **17**, 574-583 | Overlap population with datasets |
| 15. Inoue, Y., Matsumoto, H., Yamada, S., Kawai, K., Suemizu, H., Gika, M., Takanami, I., Nakamura, M., and Iwazaki, M. (2010) ATP7B expression is associated with in vitro sensitivity to cisplatin in non-small cell lung cancer. *Oncology letters* **1**, 279-282 | Different end-point |
| 16. Inoue, Y., Matsumoto, H., Yamada, S., Kawai, K., Suemizu, H., Gika, M., Takanami, I., Iwazaki, M., and Nakamura, M. (2010) Association of ATP7A expression and in vitro sensitivity to cisplatin in non-small cell lung cancer. *Oncology letters* **1**, 837-840 | Different end-point |
| 17. Nakagawa, T., Inoue, Y., Kodama, H., Yamazaki, H., Kawai, K., Suemizu, H., Masuda, R., Iwazaki, M., Yamada, S., Ueyama, Y., Inoue, H., and Nakamura, M. (2008) Expression of copper-transporting P-type adenosine triphosphatase (ATP7B) correlates with cisplatin resistance in human non-small cell lung cancer xenografts. *Oncology reports* **20**, 265-270 | Different end-point |
| 18. Kanzaki, A., Toi, M., Neamati, N., Miyashita, H., Oubu, M., Nakayama, K., Bando, H., Ogawa, K., Mutoh, M., Mori, S., Terada, K., Sugiyama, T., Fukumoto, M., and Takebayashi, Y. (2002) Copper-transporting P-type adenosine triphosphatase (ATP7B) is expressed in human breast carcinoma. *Japanese journal of cancer research : Gann* **93**, 70-77 | Different end-point |
|  |  |
